# CircSNX6 promotes proliferation, metastasis, and angiogenesis in hepatocellular carcinoma via miR-383-5p/VEGFA signaling pathway

**DOI:** 10.1038/s41598-024-58708-1

**Published:** 2024-04-08

**Authors:** Yuan Tian, Wenwen Han, Kaiji Lv, Long Fu, Xinhua Zhou

**Affiliations:** 1 Department of General Surgery, Ningbo Medical Center Lihuili Hospital, The Affiliated Lihuili Hospital of Ningbo University, Ningbo, 315100 Zhejiang China; 2 Department of Emergency, Ningbo Medical Center Lihuili Hospital, The Affiliated Lihuili Hospital of Ningbo University, Ningbo, 315100 Zhejiang China

**Keywords:** Hepatocellular carcinoma, Circular RNAs, circSNX6, miR-383-5p, VEGFA, Cancer, Liver cancer

## Abstract

The role of circular RNA (circRNAs) in hepatocellular carcinoma (HCC) has been extensively studied. Previous research has highlighted the regulatory role of circSNX6 in HCC cells and tissues. However, the precise mechanism underlying HCC progression still requires comprehensive investigation. The study initially utilized quantitative reverse transcription-polymerase chain reaction (qRT-PCR) to assess circSNX6 expression levels in HCC cell lines and tissues. Subsequently, the stability of circRNA was evaluated through Ribonuclease R and actinomycin D treatment assays. The impact of circSNX6 knockdown on proliferation, migration, invasion, and angiogenesis abilities was determined using various assays including colony formation, Transwell culture system, tube formation assay, and cell counting kit (CCK)-8 assays. Additionally, RNA immunoprecipitation chip and dual-luciferase reporter assays were employed to investigate the interactions between circSNX6 and miR-383-5p. Finally, an HCC xenograft tumor model in mice was established to assess the in vivo expression of circSNX6 and its functional role in HCC. Our findings revealed an elevated circSNX6 expression in HCC tissues, which was correlated with poor patient prognosis. Knockdown of circSNX6 suppressed HCC cell growth, invasion, metastasis, and angiogenesis. The downregulation of miR-383-5p, a target of circSNX6, significantly attenuated the tumor-suppressive effects induced by circSNX6 knockdown. Moreover, circSNX6 was found to modulate VEGFA expression by targeting miR-383-5p. The inhibition of HCC cell proliferation by miR-383-5p could be partially reversed by overexpressing VEGFA. Silencing circSNX6 also suppressed tumor formation and the metastasis of HCC cells in a mouse model. In summary, our findings suggest that circSNX6 promotes cell proliferation, metastasis, and angiogenesis in HCC by regulating the miR-383-5p/VEGFA pathway.

## Introduction

Hepatocellular carcinoma (HCC) is the most common form of primary liver cancer, accounting for about 90% of liver cancer cases^1^. While effective vaccination and antiviral treatments for hepatitis B/C virus (HBV/HCV) have reduced the incidence of HCC, other factors such as alcohol consumption and non-alcoholic fatty liver disease (NAFLD) are contributing to its increasing prevalence^1^. It is projected that the global mortality from HCC will reach one million deaths per year by 2030^2^. Early-stage HCC can be managed with treatments like liver resection, transplantation, chemoembolization, or ablation, leading to a median 5-year survival rate of over 70%^3^. However, early detection of HCC is challenging due to its lack of symptoms, often resulting in diagnosis at advanced stages with limited treatment options. Patients with advanced-stage HCC and underlying liver cirrhosis may not be eligible for aggressive treatments, leading to a lower 5-year survival rate of approximately 1–1.5 years^3^. Therefore, early diagnosis and treatment are crucial for improving patient outcomes, necessitating a deep understanding of the molecular mechanisms underlying HCC development.

Recent studies have revealed new biological insights into the progression of HCC, focusing on circRNAs^4^. Unlike other RNAs, circRNAs have a stable structure due to being covalently closed, often generated through back-splicing events^5^. These circular transcripts are frequently more abundant than their linear counterparts and exhibit a longer half-life, with tumor specificity. They can be detected in various human body fluids. Advancements in sequencing technologies and bioinformatics have uncovered critical circRNAs and their roles in malignant diseases^5^. This has highlighted the potential of circRNAs in risk assessment, screening, diagnosis, treatment selection, and therapeutic monitoring.

In a recent study, circRNA profiling reported several aberrantly expressed circRNAs in HCC tissues, including circGNAO1, circRNF180, circMERTK, and circSNX6 (hsa_circ_0031607). Among these aberrantly expressed circRNAs, circSNX6 was highly upregulated, but its specific mechanism and functions in HCC remain unknown^6^. Various databases have identified three microRNAs (hsa-miR-153-3p, hsa-miR-383-5p, and hsa-miR-873-5p) that interact with circSNX6, with miR-383-5p being down-regulated and considered a cancer suppressor in HCC^7–9^. Additionally, vascular endothelial growth factor A (VEGFA), an essential angiogenetic modulator, was reported to be upregulated in the HCC tissues^10–12^. Interestingly, TargetScan database predicts putative binding sites between miR-383-5p on VEGFA mRNA. Based on the above considerations, this work aims to investigate functional role of circSNX6 within the HCC cells, explore the interaction between circSNX6 with miR-383-5p, and examine their impact on HCC cell growth, angiogenesis, and metastasis.

## Experimental section

### Human HCC tissues

HCC and paired adjacent noncancerous liver samples were surgically obtained from 70 patients at the Affiliated Lihuili Hospital of Ningbo University. These patients had not undergone any treatment prior to surgery. Additionally, tissues were collected from 40 non-metastatic HCC cases and 30 metastatic HCC cases. The tissue specimens were then frozen in liquid nitrogen and stored at − 80 °C until analysis. Ethical approval for all experiments was obtained from the ethics committee of the Affiliated Lihuili Hospital of Ningbo University, and written informed consent was provided by all patients prior to sample collection.

### Cell culture and transfection

For in vitro investigations, human normal liver cells (L02 cell line) and human HCC cells (Huh7, HCCLM3, and Hep3B) were obtained from the Chinese Academy of Sciences (Shanghai, China). These cell lines were cultured in a high-glucose (HG)-Dulbecco’s modified Eagle’s medium (DMEM, Gibco, Waltham, USA) that was supplemented with 10% HyClone characterized fetal bovine serum (FBS, Gibco) and stored within the humid incubator maintained with 5% CO_2_ at 37 °C.

For transfection, Genomeditech Co., Ltd. (Shanghai, China) provided the sh-RNA targeting sequences for circSNX6 and scrambled RNA sequences (sh-NC). These sequences were then integrated into pGIPZ Lentiviral vectors from HonorGene, China. The resulting LV-sh-circSNX6 or LV-sh-NC vectors were introduced into 293 T cells along with packaging plasmid vectors using LipofectamineTM 3000 (Invitrogen, Carlsbad, USA) following the manufacturer's instructions. The viral supernatant was collected and then filtered through a 0.45 μm filter. HCC cells were transduced within the medium containing viral particles (Volume:volume = 1:1, viral supernatant : culture medium) fro 48 h. Transfected cells underwent puromycin (1 μg/ml) selection for 14 days to establish a stable cell line with circSNX6 knockdown.

### qRT-PCR analysis

Total RNA was extracted using TRIzol reagent (Invitrogen, 15,596,026) and then converted to cDNA with the PrimeScript II 1st Strand cDNA Synthesis Kit (TaKaRa, Tokyo, Japan) following manufacturer protocols. Subsequently, qRT-PCR reactions were carried out with specific primers and SYBR Premix Ex Taq II (TaKaRa) on the CFX96 Touch Real-time PCR detection instrument (Bio-Rad, Hercules, USA). Data analysis was performed using the 2 − ΔΔCT method against glyceraldehyde-3-phosphate dehydrogenase (GAPDH) as the endogenous control. The primer sequences used are provided below:

**Table:** 

circSNX6 :	Forward	5’-AGCACGAGTGTCTGCTGATGA-3’
	Reverse	5’-GAAATGTCCACCTGCAGAGCA-3’
SNX6 mRNA :	Forward	5’-ACAGGCCGAAACTTCCCAAC-3’
	Reverse	5’-GCTTCAGTTCTAACTCTGCCAGT-3’
GAPDH mRNA :	Forward	5’-CTGGGCTACACTGAGCACC-3’
	Reverse	5’-AAGTGGTCGTTGAGGGCAATG-3’
miR-383-5p :	Forward	5′-GGGAGATCAGAAGGTGATTGTGGCT-3′
	Reverse	5’- CAGTGCGTGTCGTGGAGT-3’
U6 :	Forward	5′-GCTTCGGCAGCACATATACTAAAAT-3′
	Reverse	5’- CGCTTCACGAATTTGCGTGTCAT-3’

### RNase R digestion and Actinomycin D treatment

Huh7 and Hep3B cells were inoculated at a density of 5 × 10^4^ cells/well in 24-well plates. After overnight incubation for proper cell attachment, the total RNA samples were extracted using TRIzol reagent. Subsequently, 5 μg of RNA samples were incubated at 37 °C in the presence or absence of RNase R (3 U/μg) for 15 min (Epicenter, Madison, USA). The resulting RNA was purified using the RNeasy MinElute Cleaning Kit (Qiagen) and analyzed through qRT-PCR. The stability of circSNX6 or its linear isoform was evaluated by exposing the cells to 2 mg/ml of Actinomycin D or dimethyl sulfoxide (DMSO, Sigma-Aldrich, 20–139, St. Louis, USA), followed by RNA extraction and qRT-PCR analysis.

### Colony formation and CCK-8 assays

To evaluate the growth and proliferation efficiencies of the selected HCC cell lines, we conducted the colony formation and CCK-8 assays. Initially, the HCC cell lines were seeded into 6-cm dishes at a density of 1 × 10^3^ cells/well and allowed to form colonies over a two-week period with regular medium changes. Subsequently, the cells underwent PBS washes, fixation with 4% paraformaldehyde (PFA), and staining with 0.4% crystal violet for 15 min. The number of clones containing more than 10 cells was manually counted, and the mean value was calculated from two replicate wells. Additionally, the CCK-8 assay (Abcam, ab228554, Cambridge, USA) was used to assess cell growth following the manufacturer's instructions. Cells were plated in 96-well plates with complete medium (100 μL) and incubated for various time periods. At indicated time point, CCK-8 working solution (20 μL) was added to each well and further incubated for 2 h at 37 °C. Absorbance values at 450 nm were then measured to determine relative cell activities using a microplate reader.

### Transwell assays

The migration and invasion abilities of HCC cell lines (Huh7 and Hep3B) were investigated using Transwell assays. Cells were seeded at a density of 5 × 10^4^ cells per well in the top chambers of (pore size, 8-μm, Costar) with/without Matrigel coating (BD Biosciences Co. Ltd., San Jose, USA). The bottom chambers contained a complete medium with 10% FBS, and the cells were incubated for 24 h (migration) or 48 h (invasion) at 37 °C with 5% CO_2_. Non-migratory or non-invasive cells in the upper chambers were removed, while those on the lower surface were fixed with 4% paraformaldehyde and stained with 0.1% crystal violet. Migrating and invading cells were then visualized under a microscope at 200 × magnification (Olympus Corp. Ltd., Tokyo, Japan).

### Tube formation assay

HUVECs obtained from American Type Culture Collection (ATCC) in Manassas, USA, were plated at a density of 4 × 10^3^ cells per well on 96-well plates coated with 60 μL of Matrigel (BD Biosciences Co. Ltd.). Subsequently, the cells were incubated with DMEM containing 10% FBS for 30 min at 37 °C. Following this, a suspension of HUVECs and tumor-conditioned medium were added after the Matrigel had dried, and the cells were incubated for 8 h at 37 °C with 5% CO_2_. The average number of capillary-like branches was then measured under a microscope (200 × , Olympus Corp. Ltd.).

### Fluorescence in situ hybridization (FISH)

Initially, HCC cell lines (Huh7 and Hep3B cells) were treated with paraformaldehyde for fixation, Triton X-100 for permeabilization before pre-hybridization. Subsequently, Cy-3-labeled circSNX6 probes were used for overnight hybridization at 37 °C in the dark. The cells were then washed with saline-sodium citrate buffer at 42 °C, followed by incubation with PBS containing Tween® and 5% bovine serum albumin at room temperature for 30 min. Finally, the signals were observed under a microscope from Olympus Corp. Ltd.

### Luciferase reporter assay

After inoculation into 24-well plates, Huh7 and Hep3B cells were co-transfected with luciferase vectors and miR-383-5p mimic or miR-NC (negative control for miTNA mimic). After 48-h transfection, the luciferase activity, specifically red firefly and green Renilla luciferase activities, was measured using a Luciferase Reporter Gene Detection Kit (Sigma-Aldrich) according to the manufacturer's instructions. The signals were finally detected using the EnSpire Perkin Elmer multi-plate reader.

#### RNA immunoprecipitation (RIP)

The Huh7 and Hep3B cells were initially lysed using the Imprint® RNA Immunoprecipitation Kit (Sigma, RIP-12RXN). RNA precipitation was performed using Magna RIP® RNA-Binding Protein Immunoprecipitation Kit (Sigma-Aldrich, 17–700) according to the manufacturer's instructions. In brief, a mixture of 1000 μL cell lysate from 1 million cells and 100 μL magnetic beads coupled with either anti-IgG or anti-Ago2 was prepared and incubated at 4 °C for 4 h. The beads were then washed 4 times using the washing buffer at 4 °C. Finally, qRT-PCR analysis was performed to quantify the immunoprecipitated RNAs.

#### Western blot (WB) analysis

WB analysis was conducted to assess the protein expression levels. Cells were lysed using RIPA buffer (KeyGen, Rockville, USA) on ice for 15 min. The quality of total protein collected was determined using the Enhanced Bicinchoninic Acid (BCA) Protein Assay Kit (Beyotime, P0010S, Shanghai, China). Subsequently, the protein samples were loaded onto a 10% SDS-PAGE gel and transferred onto a PVDF membrane (Millipore, Darmstadt, Germany). The membranes were then blocked in PBS-T with 5% bovine serum albumin for 1 h. Next, the blots were cut according to the size of target proteins prior to hybridisation with antibodies, followed by incubation with primary antibodies against VEGFA (1:1000, Abcam, ab46154) or GAPDH (1:10,000, Abcam, ab8245) overnight at 4 °C. Afterward, the membranes were incubated with an HRP-labeled secondary antibody (1:10,000, Invitrogen, 61–6520) for 1 h at room temperature. Following washing steps, an enhanced chemiluminescence (ECL) kit (Sigma) was applied to develop protein signals on the PVDF membranes. Protein bands were visualized and analyzed using ImageJ software.

#### Flow cytometric detection of apoptosis

NSCLC cells were suspended at a density of 1 × 10^6^ cells/mL in medium, and washed twice with PBS and centrifuged at 3000 rpm for 10 min. Cell pellet was re-suspended in 1 mL staining buffer containing 5 μL of Annexin V-FITC and 5 μL of propidium iodide (PI) for 15-min incubation at 4 °C in the dark. Stained cells were analyzed for apoptosis on the CytoFLEX flow cytometer (Beckman Coulter Inc., Brea, USA).

#### Nude mouse model of xenograft

To investigate the results in vivo, we conducted subcutaneous xenograft experiments using 4–5-week-old male BALB/c nude mice obtained from Charles River (Beijing, China). Initially, Huh7 cells were stably infected with lentivirus carrying either sh-NC or sh-circSNX6. The cells were subcutaneously inoculated into the right flank of the mice at 5 × 10^6^ cells per animal. For the metastasis model, 2 × 10^6^ HCC cells were injected into nude mice via the tail vein. Tumor volumes were measured every 7 days, with final tumor weight recorded at the end of the study. After 4 weeks, the mice were euthanized through cervical dislocation, and lung tissue was collected for hematoxylin–eosin (H&E) staining to assess metastasis. Micro-metastatic lesions were examined using a dissecting microscope. Animal experimental procedures in the study gained the approval from Animal Ethics Committee of Lihuili Hospital of Ningbo University.

#### Statistical analysis

The experimental data were reported as mean ± standard deviation (S.D.). Data analysis was conducted using Prism software (Graph Pad Software 7.0), with continuous variables compared using either Student's *t*-test or the Wilcoxon test, as deemed appropriate. Survival curves were generated using the Kaplan–Meier method, and the results were assessed using the Log-rank test. All data were deemed statistical significant when *P* < 0.05.

## Results

### circSNX6 is highly expressed in HCC cells and tissues

**Figure 1:**
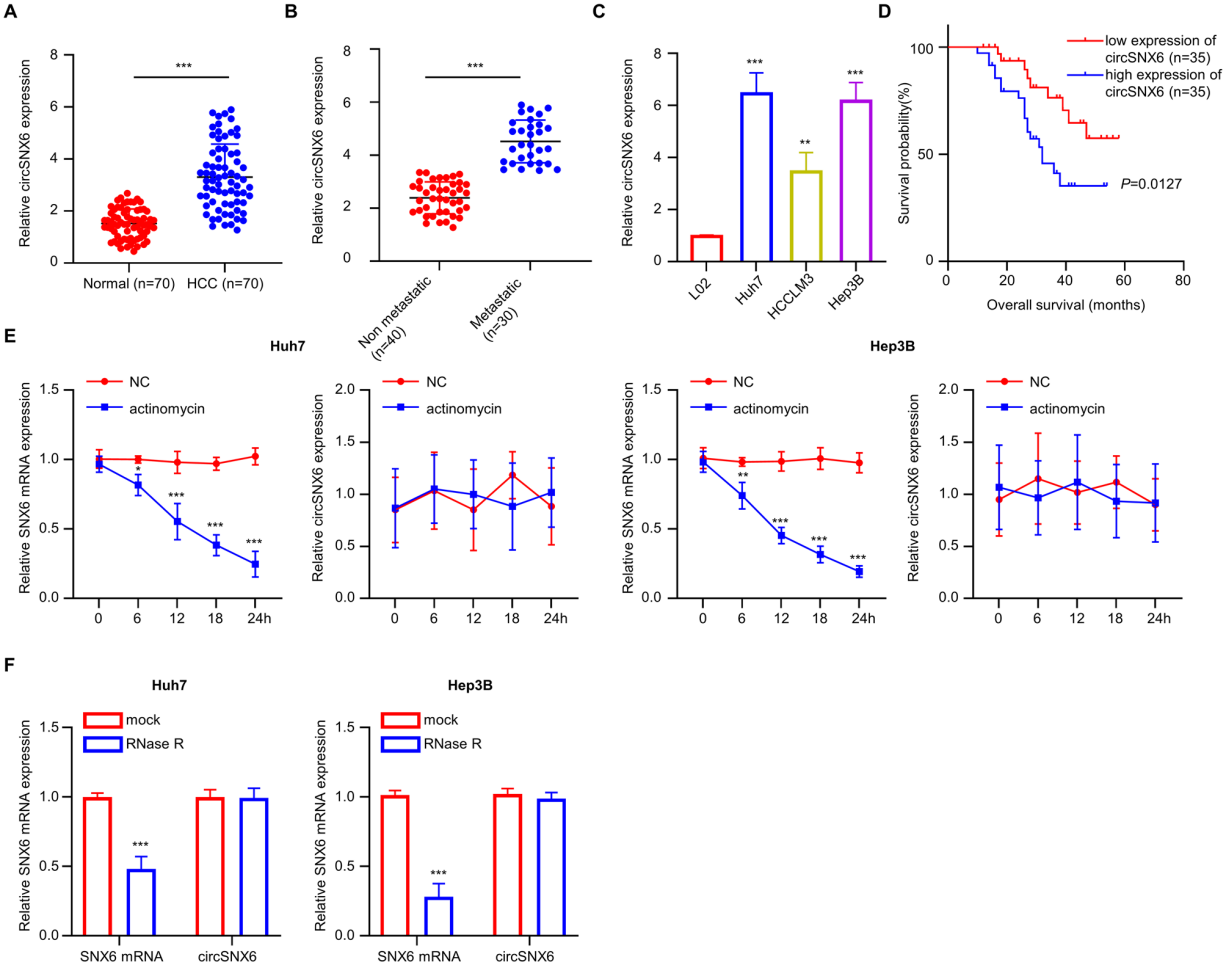
circSNX6 is highly expressed in HCC cells and tissues. qRT-PCR analysis shows (**A**) the expression levels of circSNX6 within 70 pairs of HCC and matched normal tissues (*** indicates *P* < 0.001), (**B**) circSNX6 levels within metastatic (*n* = 30) and non-metastatic tissues (*n* = 40), and (**C**) circSNX6 expression within HCC cells (Huh7, HCCLM3, and Hep3B) and normal liver cells (L02). (**D**) The KM plot presents the association between circSNX6 expression level and the overall survival of HCC patients (high *vs.* Low expression groups). qRT-PCR analysis shows (**E**). the relative expression of SNX6 mRNA and circSNX6 in Huh7 and Hep3B cells after Actinomycin D treatment, and (**F**) the relative levels of SNX6 mRNA and circSNX6 in Huh7 and Hep3B cells after RNase R treatment. Summary from 3 dependent experiments. ** indicates *P* < 0.01, and *** designates *P* < 0.001.

The expression levels of circSNX6 were initially analyzed in 70 pairs of HCC and paracancerous tissue samples, as well as HCC cell lines, using qRT-PCR analysis. The results indicated a significantly higher level of circSNX6 expression in the HCC tissues compared to the normal specimens (*P* < 0.01, Fig. 1A). Furthermore, when comparing the relative circSNX6 expression levels between metastatic (*n* = 30) and non-metastatic tissues (*n* = 40), it was found that circSNX6 expression was notably higher in the metastatic group (*P* < 0.01; Fig. 1B). Additionally, the circSNX6 levels were significantly elevated in the HCC cell lines (Huh7, Hep3B, and HCCLM3) compared to the normal liver cells (L02 cell line) (*P* < 0.01, Fig. 1C). The high circSNX6 expression in HCC patients was found to be correlated with advanced TNM stage (*p* = 0.022), tumor size (*p* = 0.003), HCV positivity (*p* = 0.02), and metastasis (*p* = 0.00) (Table 1).

**Table 1:** Correlative analysis of circSNX6 expresion with clinicopathological features.

Characteristic	circSNX6 expression		*P* value
	Low (*n* = 35)	High (*n* = 35)	
Age, years (mean ± SD)	61.7 ± 11.0	60.85 ± 13.65	0.7751
Gender			0.631
Male	17	15	
Female	18	20	
Etiology			0.626
HBV	13	15	
Non-HBV	22	20	
Stage			0.022
I + II	7	16	
III	28	19	
PHT			0.051
Negative	10	18	
Positive	25	17	
AFP, ng/mL			0.334
< 400	13	17	
≥ 400	22	18	
Tumor size (cm)			0.003
< 5	29	18	
> 5	6	17	
Tumor numbers			0.255
Solitary	10	6	
Multiple	25	29	
Portal vein invasion			0.089
No	17	24	
Yes	18	11	
Metastasis			0.000
Without metastasis	23	7	
Metastasis	12	28	
HCV			
Yes	8	21	0.02
No	27	14	
Cirrhosis			
Yes	15	17	0.230
No	20	18	

To investigate the prognostic significance of circSNX6 in HCC, we analyzed the correlation between circSNX6 expression and overall survival rates using the Kaplan–Meier method. High circSNX6 expression was associated with worse overall survival in HCC cases compared to those with low expression (*p* = 0.0127, Fig. 1D). Additionally, we assessed circSNX6 stability by treating HCC cells (Huh7 and Hep3B cell lines) with actinomycin D and observed changes in circSNX6 expression levels over a 24-h time course. While SNX6 mRNA levels decreased significantly in both cell lines, circSNX6 expression levels remained unchanged (*P* < 0.001, Fig. 1E). Similarly, treatment with RNase R significantly decreased SNX6 mRNA expression but had no effect on circSNX6 levels in both cell lines (*P* < 0.001, Fig. 1F). These findings suggest that circSNX6 forms a closed-loop structure, as evidenced by its resistance to actinomycin D and RNase R exonuclease. Overall, our results indicate an elevated circSNX6 expression in HCC cell lines and tissues, which may serve as a potential prognostic marker for HCC patients.

### circSNX6 silencing inhibits HCC cell growth, migration, invasion, and angiogenesis

**Figure 2:**
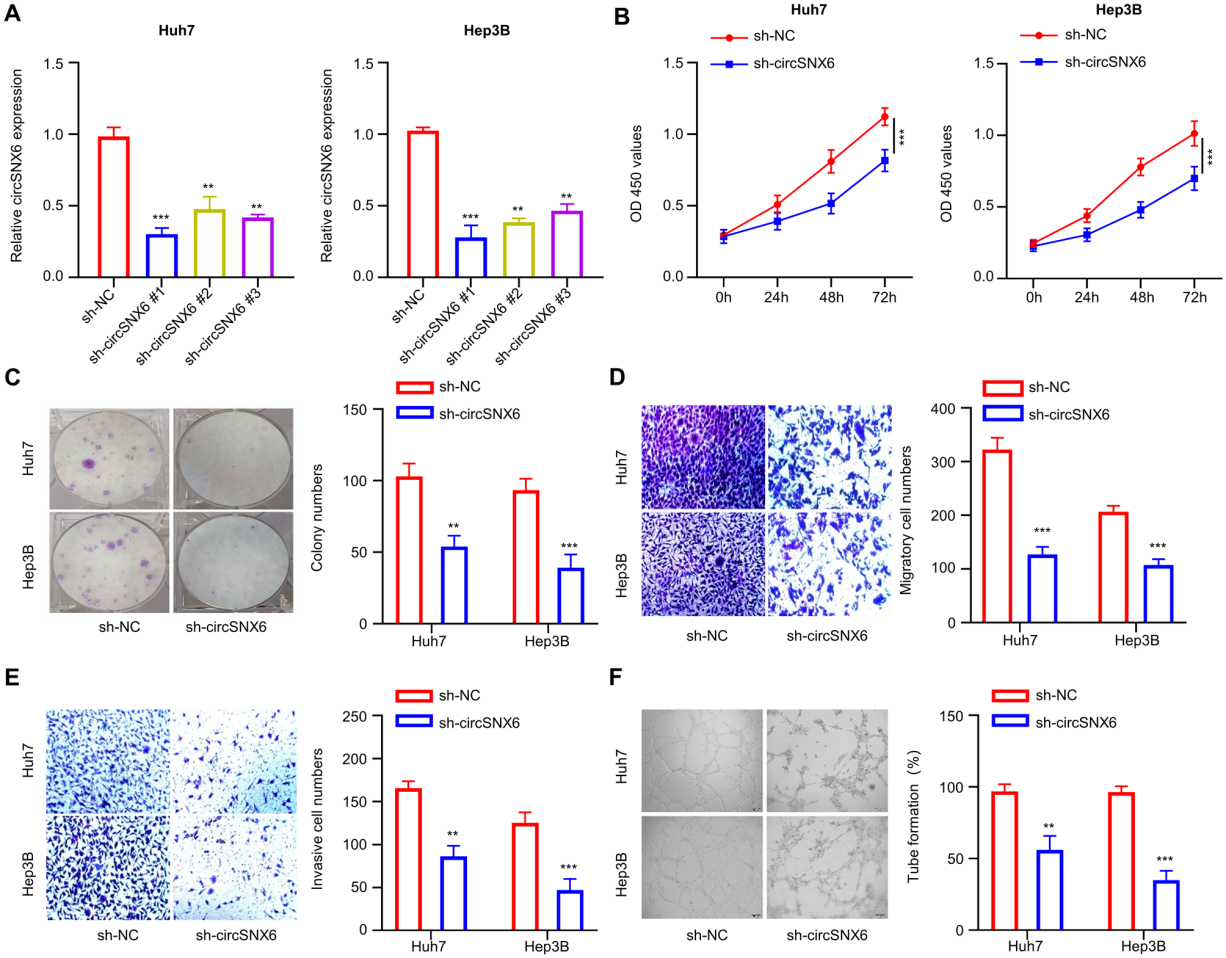
circSNX6 silencing inhibits HCC cell growth, migration, angiogenesis, and invasion. (**A**) qRT-PCR analysis shows the relative circSNX6 expression within Hep3B and Huh7 cells after the introduction of sh-circSNX6#1, sh-circSNX6#2, sh-circSNX6#3, or sh-NC. (**B**) CCK-8 assay shows the relative cell growth at 0, 24, 48, and 72 h in Huh7 and Hep3B cells with sh-NC or sh-circSNX6#1. (**C**) The colony formation assays in Huh7 and Hep3B cells with sh-NC or sh-circSNX6#1. (**D**) Transwell migration (**E**) and invasion assay in Huh7 and Hep3B cells with sh-NC or sh-circSNX6#1. (**F**) Tube formation assay in HUVECs cultured with the conditioned medium from Huh7 or Hep3B cells with sh-NC or sh-circSNX6#1. Summary from 3 dependent experiments. * specifies *P* < 0.05, ** indicates *P* < 0.01, and *** designates *P* < 0.001.

To investigate the role of circSNX6 in HCC progression, HCC cell lines (Huh7 and Hep3B) with high circSNX6 expression levels (Fig. 1C) were selected to stably express sh-NC or sh-circSNX6. qRT-PCR results demonstrated a significant decrease of circSNX6 expression in sh-circSNX6 expressing Huh7 and Hep3B cell lines compared to the control group (sh-NC, Fig. 2A, P < 0.01), confirming effective circSNX6 knockdown. Cells expressing sh-circSNX6#1 was selected for the following functional assays. Subsequent colony formation and CCK-8 assays showed that circSNX6 silencing markedly inhibited the growth of HCC cell lines (Fig. 2B,C,* P* < 0.01). Additionally, transwell assays revealed a notable decrease in the migratory and invasive cell number after circSNX6 knockdown, suggesting that circSNX6 knockdown undermines the migration and invasion abilities of HCC cell lines (Fig. 2D,E,* P* < 0.01). Furthermore, downregulation of circSNX6 in Hep3B and Huh7 cells impaired the tube formation in human umbilical vascular endothelial cells (HUVECs, Fig. 2F,* P* < 0.01).

### miR-383-5p is identified as a miRNA target of circSNX6

**Figure 3:**
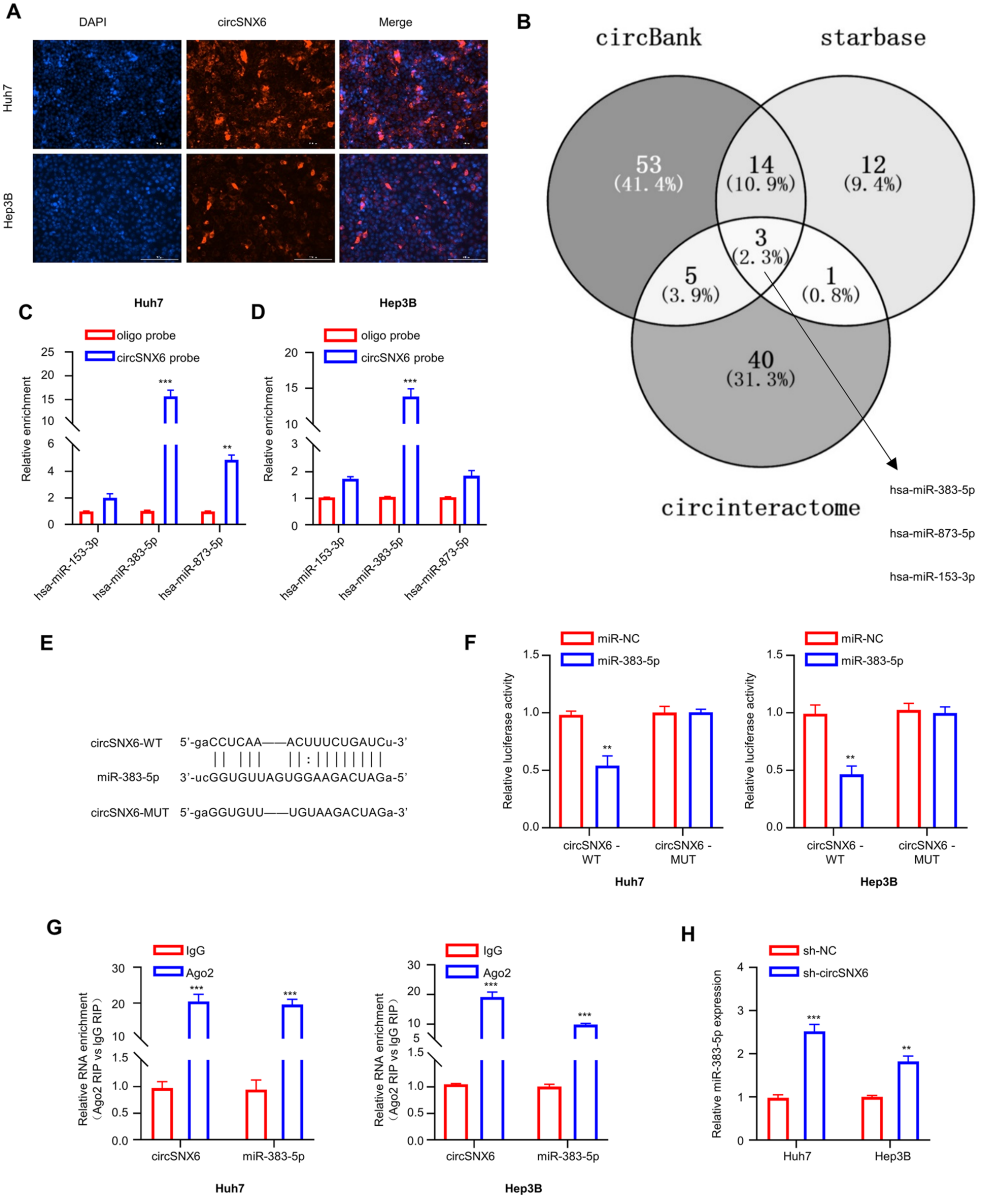
miR-383-5p is a miRNA target of circSNX6. (**A**) FISH staining of the localization of circSNX6 in Huh7 and Hep3B cells. (**B**) The Venn diagram shows the predicted miRNA targets of circSNX6 by three databases. (**C**-**D**) RNA precipitation analysis using control or circSNX6 probe in Huh7 and Hep3B cells. The precipitated miRNA was analyzed using qRT-PCR. (**E**) The image shows shows the putative binding sites (WT) or mutated binding sequences (MUT) between circSNX6 and hsa-miR-383-5p. (**F**) Luciferase activity measurement in Huh7 and Hep3B cells after WT or MUT circSNX6 reporter was co-transfected with miR-NC or miR-383-5p mimic. (**G**) RIP-qPCR analysis of circSNX6 and miR-383-5p precipitated by Ago2 or nonspecific rabbit IgG in Hep3B and Huh7 cells. (**H**) qRT-PCR analysis of miR-383-5p expression levels in Hep3B and Huh7 cells with or without circSNX6 silencing. Summary from 3 dependent experiments. ** indicates *P* < 0.01, and *** designates *P* < 0.001.

Prior to identifying the miRNA target of circSNX6, we first detected the intracellularly expressed circSNX6 using fluorescence in-situ hybridization (FISH) analysis. It was observed that circSNX6 was predominantly located within the cytoplasm of Huh7 and Hep3B cell lines (Fig. 3A). Subsequently, we utilized three databases—StarBase (http://starbase.sysu.edu.cn/), circBank (http://www.circbank.cn/), and circInteractome (https://circinteractome.nia.nih.gov/) to predict potential interactions between miRNAs and circSNX6. Notably, three common miRNAs were identified: hsa-miR-153-3p, hsa-miR-383-5p, and hsa-miR-873-5p (Fig. 3B). qRT-PCR analysis demonstrated that circSNX6 probe could efficiently precipitate miR-383-5p in both Huh7 and Hep3B cell lines (Fig. 3C,D). The putative binding sites between circSNX6 and miR-383-5p, as well as the mutated sequences was illustrated in Fig. 3E. To validate the bindings sites, luciferase reporter vectors containing mutated (MUT) or wild-type (WT) miR-383-5p target sequences were transfected into Huh7 and Hep3B cell lines. The luciferase reporter activities were notably reduced by miR-383-5p mimic in cells transfected with the WT sequence compared to the MUT sequence (Fig. 3F), indicating the interaction between miR-383-5p and circSNX6 via the WT sequences. Subsequently, an RIP assay using argonaute 2 (Ago2) antibody was performed in Huh7 and Hep3B cell lines, which showed that both circSNX6 and miR-383-5p were significantly enriched in the Ago2 RIP samples compared to the IgG control samples, indicating the physical association between circSNX6 and miR-383-5p (*P* < 0.001, Fig. 3G). Furthermore, Hep3B and Huh7 cells with circSNX6 silencing exhibited elevated levels of miR-383-5p compared to sh-NC control (*P* < 0.01, Fig. 3H). Collectively, these experimental results support the direct interaction between circSNX6 and miR-383-5p in HCC cell lines.

### Downregulating miR-383-5p attenuates the effect of circSNX6 knockdown in HCC cells

**Figure 4:**
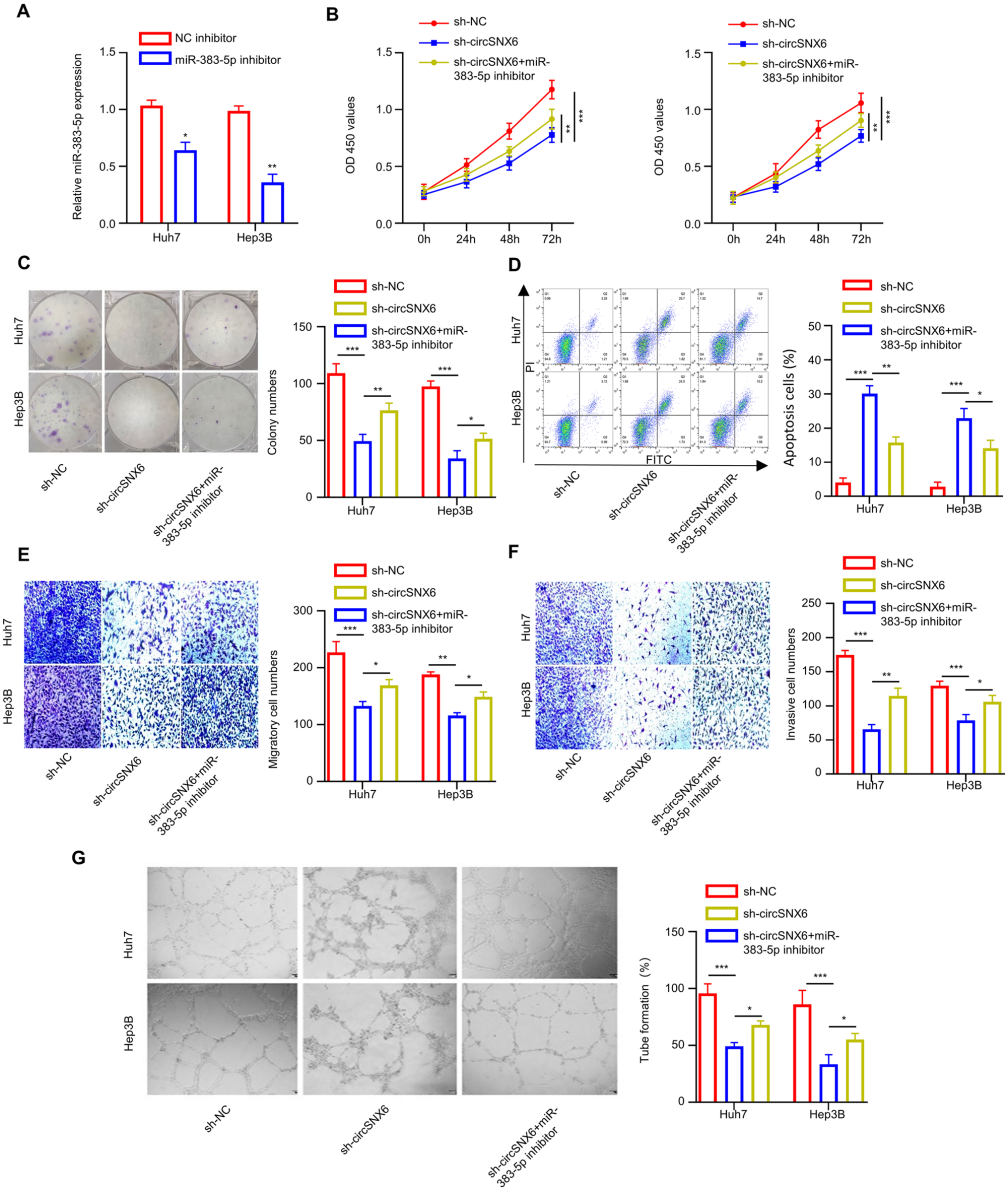
Downregulating miR-383-5p partially rescued the effect of circSNX6 knockdown-induced inhibition on HCC cells. (**A**) qRT-PCR analysis shows the relative miR-383-5p levels in Huh7 and Hep3B after the transfection with inhibitor-NC (negative control) or miR-383-5p inhibitor. (B-G) Hep3B and Huh7 cells were divided into the following groups: sh-NC, sh-circSNX6, or sh-circSNX6 + miR-383-5p inhibitor. (**B**) CCK-8 cell growth assay; (**C**) Colony formation experiment; (**D**) Apoptosis detection by flow cytometer; (**E**) Transwell migration assay; (**F**) Transwell invasion assay; and (**G**) Tube formation assay HUVECs using the conditioned medium. Summary from 3 dependent experiments. * specifies *P* < 0.05, ** indicates *P* < 0.01, and *** designates *P* < 0.001.

Next, Huh7 and Hep3B cells were transfected with a miR-383-5p inhibitor to investigate the impact on circSNX6 knockdown. There was a significant reduction in miR-383-5p expression following the inhibitor transfection (*P* < 0.01, Fig. 4A). Both CCK-8 and colony formation assay results revealed that the suppression on the proliferation of HCC cells by circSNX6 knockdown was partially rescued by miR-383-5p inhibitor (*P* < 0.01, Fig. 4B,C). Flow cytometry analysis demonstrated that circSNX6 knockdown induce apoptosis in HCC cells, and the introduction of miR-383-5p inhibitor suppressed this effect (*P* < 0.001, Fig. 4D). Furthermore, Transwell assay results showed a reduction in migration and invasion abilities upon circSNX6 silencing, which was partially reversed by miR-383-5p inhibitor (*P* < 0.01, Fig. 4E,F). Similarly, the tube formation ability of HUVEC cells were also increased when cultured in the medium from HCC cells with miR-383-5p inhibitor treatment (*P* < 0.01, Fig. 4G). These findings indicate that downregulating miR-383-5p attenuates the effect of circSNX6 knockdown in HCC cells.

### circSNX6 modulates VEGFA expression via targeting miR-383-5p

**Figure 5:**
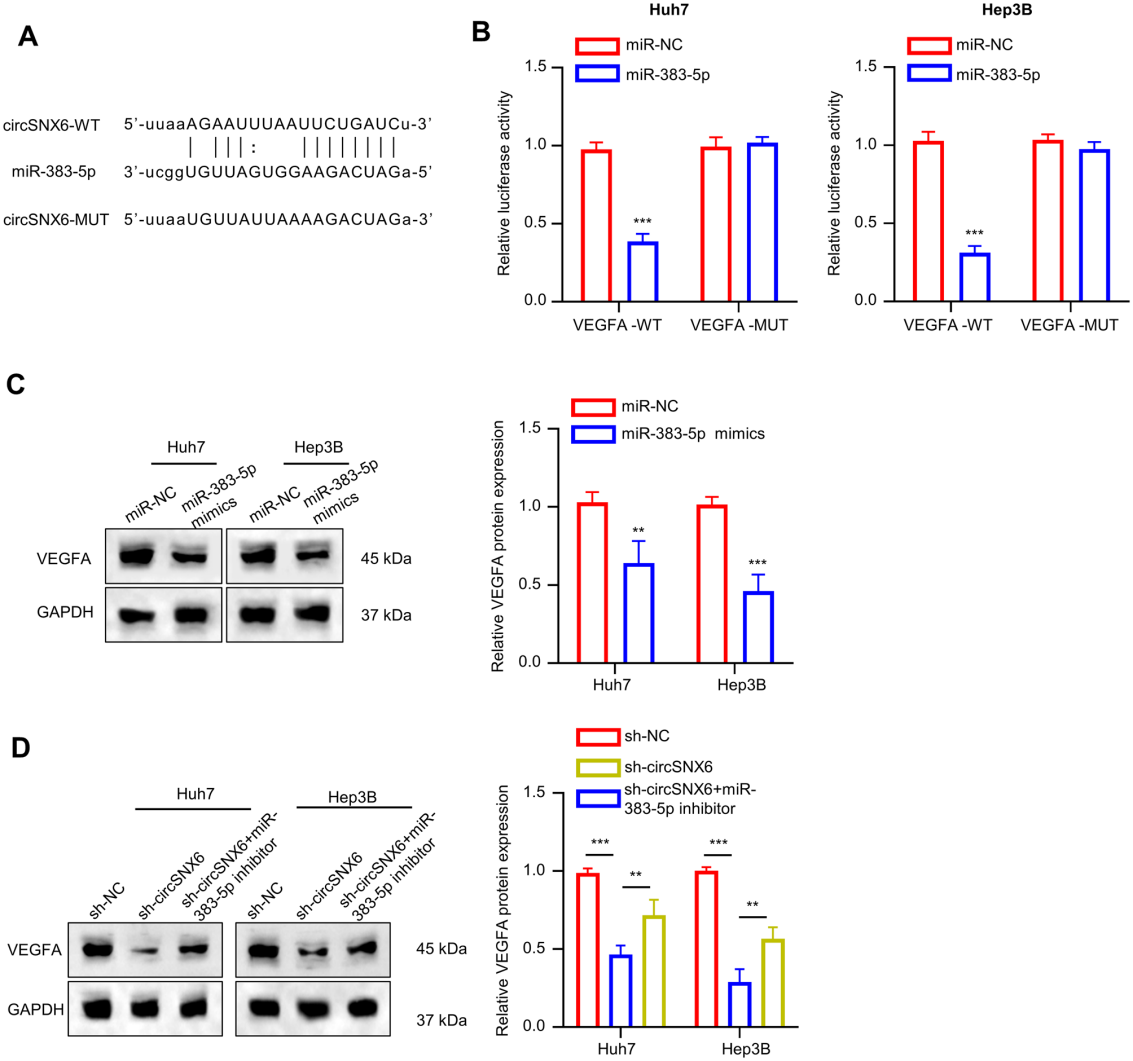
circSNX6 regulates VEGFA expression via targeting miR-383-5p. (**A**) The image shows the predicted miR-383-5p target sequences (WT) and mutated sequences (MUT) within VEGFA mRNA 3’-UTR. (**B**) Luciferase activity assay in Huh7 and Hep3B cells after transfection with MUT or WT VEGFA reporter plasmid and miR-NC or miR-383-5p mimic. (**C**) WB analysis of VEGFA protein levels within Huh7 and Hep3B cells after transfection with miR-383-5p mimics or miR-NC. (**D**) WB analysis of VEGFA protein levels within Huh7 and Hep3B cells of sh-NC, sh-circSNX6, or sh-circSNX6 + miR-383-5p inhibitor groups. Summary from 3 dependent experiments. ** indicates *P* < 0.01, and *** designates *P* < 0.001.

The potential mechanistic role of miR-383-5p in HCC cell lines was explored using the starBase database, which predicted VEGFA as a potential target of miR-383-5p. Figure 5A illustrates the predicted interacting sequences of miR-383-5p in the 3’-UTR of VEGFA. A luciferase reporter vector assay with MUT or WT VEGFA 3’ UTR was conducted to confirm the interaction between miR-383-5p and VEGFA. Co-transfection with miR-383-5p mimic resulted in significantly reduced of luciferase activities in WT VEGFA reporter, while no inhibition was observed with MUT VEGFA reporter (*P* < 0.001, Fig. 5B). Furthermore, WB analysis showed a significant decrease in VEGFA protein expression in cells transfected with miR-383-5p mimic compared to the negative control group (*P* < 0.01, Fig. 5C). Cells with sh-circSNX6 expression also exhibited reduced VEGFA protein expression, which was reversed when miR-383-5p inhibitor was introduced (*P* < 0.01, Fig. 5D). These results suggest that VEGFA is a target of miR-383-5p and that circSNX6 can modulate VEGFA expression in HCC cells by regulating miR-383-5p activity.

### VEGFA overexpression partially reverses miR-383-5p-induced inhibition in HCC cells

**Figure 6:**
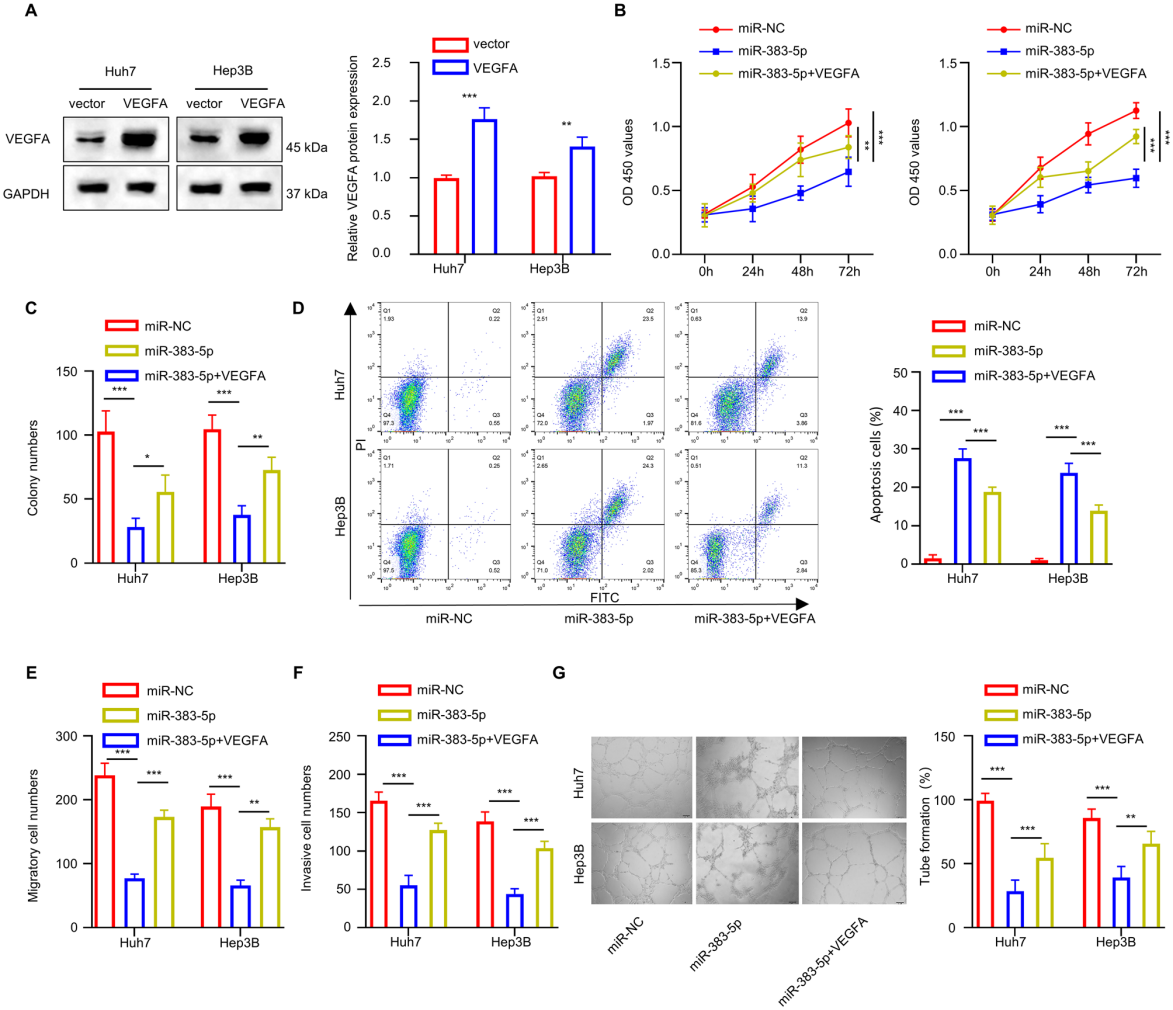
VEGFA overexpression partially reverses miR-383-5p-induced inhibition on HCC cells. (**A**) WB analysis shows the VEGFA overexpression within Hep3B and Huh7 cells after the trasfection of VEGFA expression vector. (B-G) Huh7 and Hep3B cells were divided into the following groups: miR-NC, miR-383-5p and miR-383-5p + VEGFA. (**B**) CCK-8 cell growth assay; (**C**) Colony formation experiment; (**D**) Apoptosis analysis by flow cytometer; (**E**) Transwell migration assay; (**F**) Transwell invasion assay; and (**G**) Tube formation assay HUVECs using the conditioned medium.. Summary from 3 dependent experiments. * specifies *P* < 0.05, ** indicates *P* < 0.01, and *** designates *P* < 0.001.

We further reported an effect of VEGFA overexpression on miR-383-5p-induced inhibition in HCC cells. Huh7 and Hep3B cells exhibited significantly higher VEGFA protein expression when transfected with VEGFA expression vector (*P* < 0.01, Fig. 6A). Subsequently, Hep3B and Huh7 cells were transfected with miR-NC, miR-383-5p mimic, or miR-383-5p + VEGFA vector. miR-383-5p mimic transfection resulted in a notable decrease in cell proliferation compared to the miR-NC group. Forced VEGFA expression showed a rescue effect on cell proliferation (*P* < 0.01, Fig. 6B,C). Accordingly, apoptosis induced by miR-383-5p mimic was suppressed when VEGFA was overexpressed (*P* < 0.001, Fig. 6D). Furthermore, increased miR-383-5p expression also reduced the migration and invasion in Hep3B and Huh7 cells, and suppressed the angiogenesis in HUVECs. These effects were partially rescued by VEGFA overexpression (*P* < 0.01, Fig. 6E,G). These results suggest an functional interaction between miR-383-5p and VEGFA in dictating the malignancy of HCC cells.

### Knockdown of circSNX6 inhibits HCC progression in vivo

**Figure 7:**
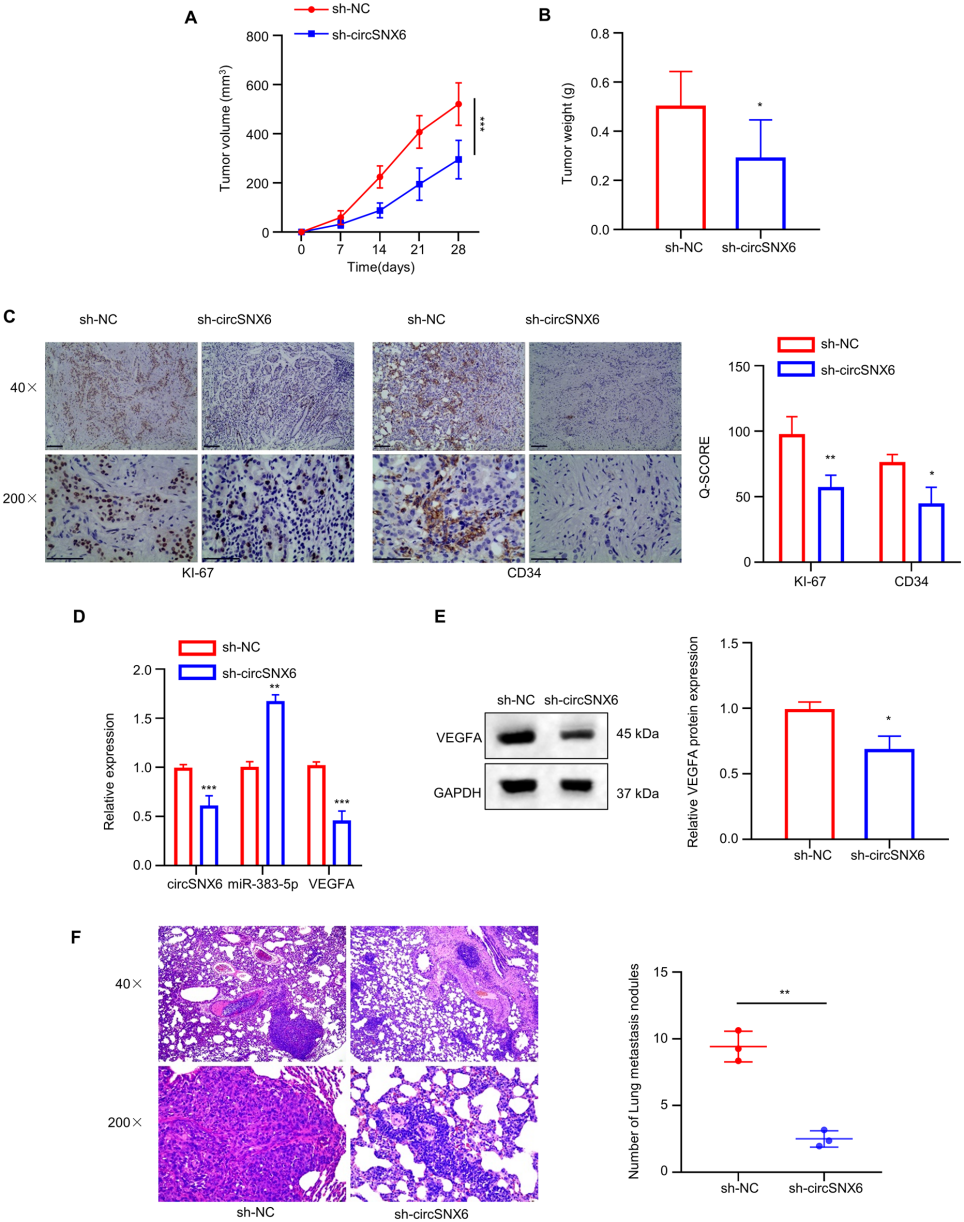
Knockdown of circSNX6 inhibits HCC progression in vivo. (**A**) The growth curve xenograft tumor volume and (**B**) the tumor weight of sh-NC and sh-circSNX6 Huh7 cells in nude mice. (**C**) KI-67 and CD34 staining in the xenograft tumor samples in sh-NC and sh-circSNX6 groups. (**D**) qRT-PCR analysis of circSNX6, miR-383-5p, and VEGFA in tumor tissues. (**E**) WB analysis of VEGFA protein levels in tumor tissues. (**F**) H&E staining of the metastatic lung nodules in sh-NC and sh-circSNX6 groups. *n* = 5 animals in each group. * specifies *P* < 0.05, ** indicates *P* < 0.01, and *** designates *P* < 0.001.

After conducting initial investigations in vitro, we proceeded to study the impact of circSNX6 on the tumor formation and metastasis of HCC cells in vivo. To this end, a xenograft model was established in BABL/c nude mice through subcutaneous cell inoculation. Huh7 cells with circSNX6 silencing exhibited significantly reduced tumor weight and volume compared to the sh-NC group (Fig. 7A,B,* P* < 0.05). Immunohistochemical (IHC) analysis demonstrated decreased levels of Ki-67 (cell proliferation marker) and CD34 (angiogenesis marker) in sh-circSNX6 group (Fig. 7C,* P* < 0.05). Moreover, circSNX6 knockdown led to decreased levels of circSNX6 and VEGFA expression, but increased miR-383-5p levels in the xenograft tumor samples (*P* < 0.05, Fig. 7D). WB analysis confirmed a significant reduction in VEGFA expression in xenograft tumor with circSNX6 silencing (*P* < 0.05, Fig. 7E). In the mouse model of metastasis with tail vein injection, circSNX6 silencing also notably decreased the rate of lung metastasis, with fewer metastatic lung nodules observed in the sh-circSNX6 group compared to the sh-NC group (*P* < 0.05, Fig. 7F). Overall, these results indicate that downregulation of circSNX6 effectively inhibits the progression of HCC in vivo.

## Discussion

The discovery of aberrantly expressed circRNAs in HCC over the past few decades has significantly advanced our understanding of the molecular pathogenesis and heterogeneity of metabolic diseases, particularly cancer^6,13,14^. One such circRNA, circSNX6, has been identified as being regulated in various cancers, including HCC, although its exact role in tumorigenesis remains unclear^6^. In this work, we investigated the expression pattern of circSNX6 and its function role in the cell and animal models of HCC. The results showed that circSNX6 was over-expressed in HCC cells and tissues, correlating with poor patient prognosis. Knocking down circSNX6 significantly inhibited the growth, invasion, metastasis, and angiogenesis of HCC cells. The tumor-suppressing effect could be partially reversed by modulating miR-383-5p or VEGFA expression. Additionally, a xenograft tumor model was established to further validate these findings in vivo, demonstrating that circSNX6 plays a crucial role in HCC progression by interacting with miR-383-5p and VEGFA.

Previous research has shown that circRNAs with tumor-suppressing properties are often decreased, while those with pro-tumorigenic functions are commonly upregulated in cancer. In HCC, specific circRNAs such as circRNF180, cSMARCA5, circ_0051443, circMTO1, and circTRIM33-12 have been identified as tumor suppressors, and their suppression seems to facilitate HCC progression^6,15–18^. On the other hand, several oncogenic circRNAs (circRHOT1, circMAT2B, circASAP1, circMET, circ-LRIG3, and circRNA_104348) have been recognized to promote tumor growth and metastasis in HCC^19–25^. These studies have highlighted the impact of dysregulated circRNAs on cancer development and treatment response in HCC. For instance, upregulated circUHRF1 has been associated with inducing natural killer (NK) cell dysfunction, potentially causing resistance to anti-PD-1 therapy in HCC^26^. Our findings suggest that circSNX6 may function as an oncogenic factor in HCC, indicating that targeting circSNX6 can be developed as an intervention to impede the malignant progression of HCC. Despite the increasing focus on abnormal circRNA expression in cancer, there is still limited understanding of the underlying mechanisms driving these variations. A previous study revealed that RNA-binding protein 3 plays a role in promoting HCC cell proliferation through the generation of SCD-circRNA 2, underscoring the importance of RNA-binding proteins in circRNA formation^27^. Nevertheless, further validation in larger and independent cohorts is necessary due to potential variability in circRNA expression among different patient groups. Future research efforts should aim to elucidate the specific functions of individual circRNAs and explore the factors contributing to their dysregulated expression in HCC.

The interactions between circRNA, miRNA, and mRNA are frequently implicated in various cancers due to their involvement in the initiation and progression of the disease^28–30^. Previous studies have shown that circRNAs can act as oncogenes or tumor suppressors by sequestering miRNAs and inhibiting their activity^31^. This interaction allows a single circRNA to bind to one or more miRNAs, indirectly affecting the expression of corresponding mRNAs^31^. Our study indicates that circSNX6 plays an oncogenic role in the regulation of HCC. Specifically, inhibiting miR-383-5p promotes HCC cell growth, invasion, metastasis, and angiogenesis, while increasing miR-383-5p levels reduces the expression of a key angiogenic factor VEGFA, suggesting a suppressive role of miR-383-5p in HCC development. The upregulation of circSNX6 may lead to decreased miR-383-5p expression in HCC cell lines and tissues. These findings suggest that the interaction between circSNX6 and miR-383-5p is crucial in the regulation of HCC progression.

The abnormal expression of circRNAs has been linked to malignant transformation^5^ This study revealed that circSNX6 actively promotes the proliferation, migration, invasion, and angiogenesis of HCC cells in both the cell and in animal models. HCC is known for its high vascularity, with angiogenesis playing a critical role in its progression, invasion, and spread^32^ Angiogenesis involves the increased production of growth factors like VEGFA, which enhance blood vessel formation during cancer growth. The study highlighted the relationship between circSNX6 and VEGFA, a key vascular growth factor. Knocking down circSNX6 led to a notable decrease in VEGFA levels, indicating that circSNX6 may regulate VEGFA expression in HCC cells. Additionally, VEGFA mRNA contains a binding site for the tumor suppressor miR-383-5p. Overexpressing VEGFA partially counteracted the inhibitory effect of miR-383-5p on HCC cells, suggesting that miR-383-5p plays a role in the circSNX6-mediated modulation of VEGFA expression. In the context of HCC, targeting angiogenic factors and their regulatory networks through circRNAs could be a promising approach for HCC management.

## Conclusion

In summary, we reported that HCC cell line and tissues exhibited elevated levels of circSNX6, and circSNX6 upregulation is linked with a dismal prognosis in HCC patients. Notably, experimental findings on proliferation, invasion, and migration capabilities indicated that circSNX6 potentially plays a significant role in promoting the growth, angiogenesis, and the metastasis of HCC cells. Our findings also indicate that targeting circSNX6 can be developed as an intervention to impede the malignant progression of HCC. Despite the increasing interest, the mechanisms underlying the deregulation of circRNAs in various cancer types remains largely unexplored. Furthermore, a specific panel of deregulated genes have been demonstrated with prognostic values HCC^33^. Therefore, it is crucial to elucidate how dysregulated circRNAs function independently and in concert to regulate specific targets in HCC. It is surmised that circSNX6, together with our deregulated circRNAs, may serve as prognostic signature for HCC, which needs to be validated in the future study (Supplementary Figures).

### Ethics approval

All the experiments gained approval from the ethics committee of the Affiliated Lihuili Hospital of Ningbo University. All procedures performed in studies involving human participants were in accordance with the ethical standards of the institutional and/or national research committee and with the 1964 Helsinki declaration and its later amendments or comparable ethical standards.

Each animal experimental procedure gained approval from Animal Ethics Committee of Lihuili Hospital of Ningbo University. The experimental protocol was performed in accordance with the relevant guidelines and regulations of the Basel Declaration. The study is reported in accordance with ARRIVE guidelines (https://arriveguidelines.org).

### Supplementary Information

Supplementary Figures.

## Data Availability

The datasets used and/or analyzed during the current study are available from the corresponding author via email request.
