# Supplementary figures and images for: CircSNX6 promotes proliferation, metastasis, and angiogenesis in hepatocellular carcinoma via miR-383-5p/VEGFA signaling pathway
Source: Sci Rep. 2024 Apr 8;14:8243. doi: 10.1038/s41598-024-58708-1 (PMC11001896; doi:10.1038/s41598-024-58708-1)

fig5C

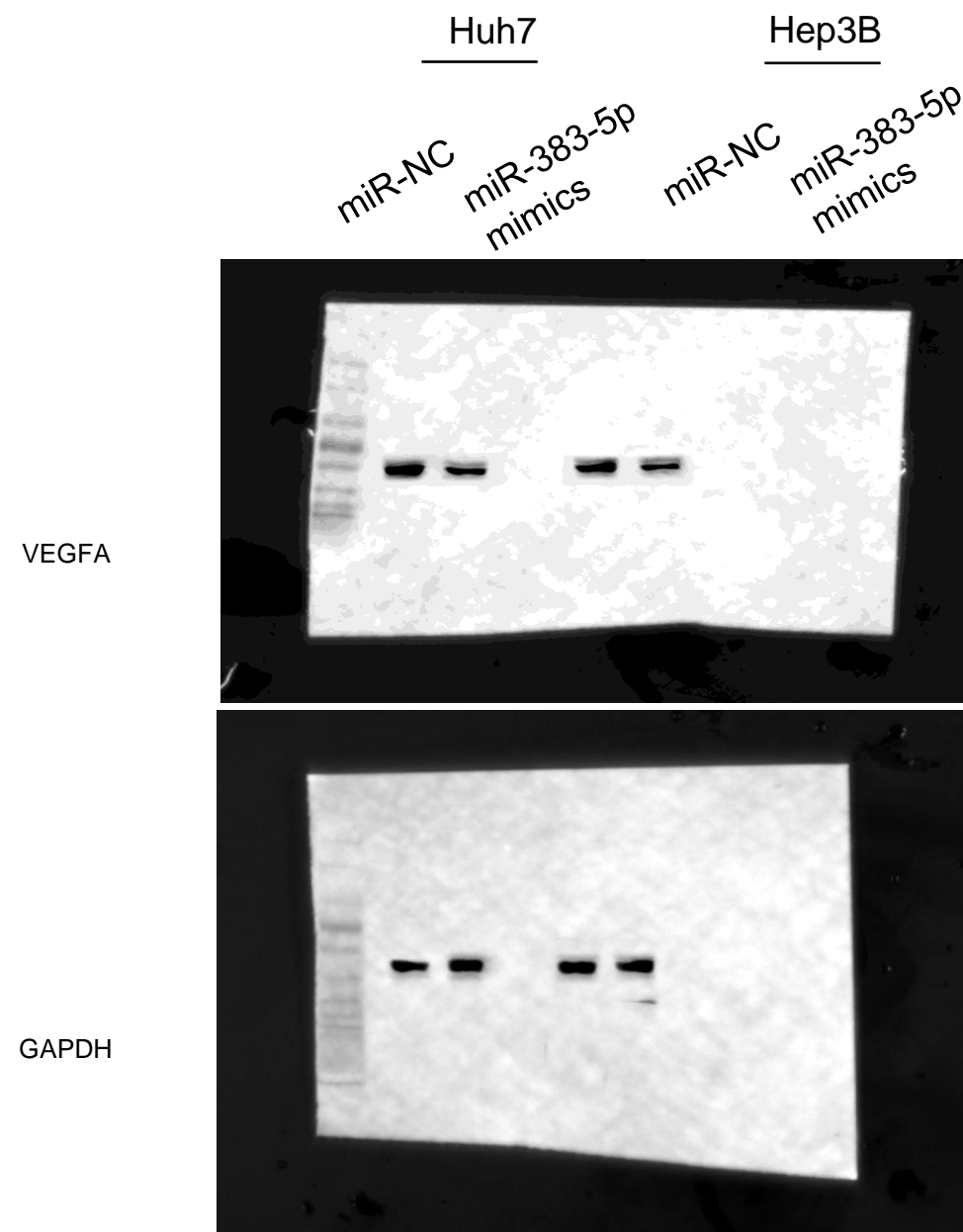

fig5D

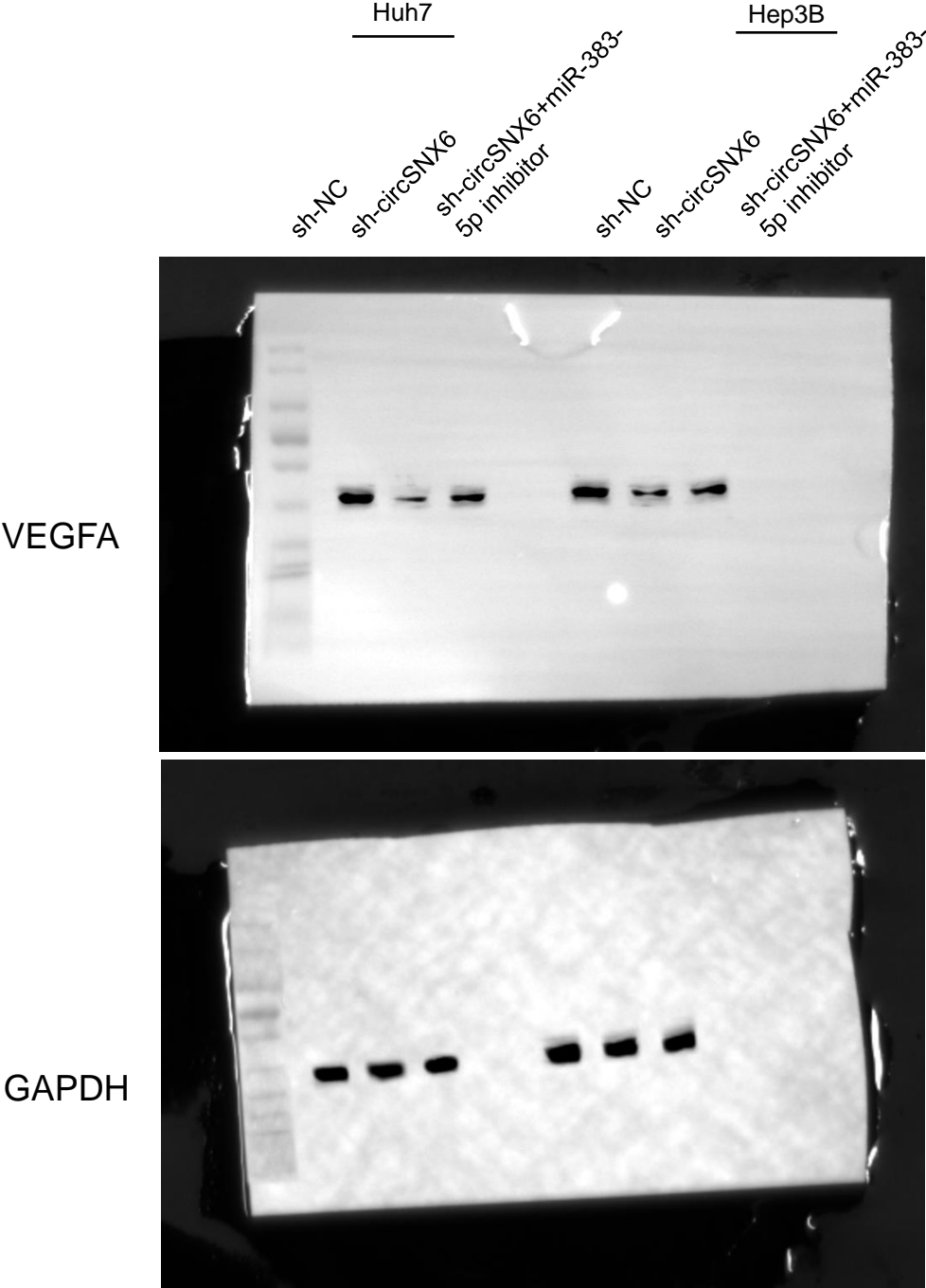

fig6A

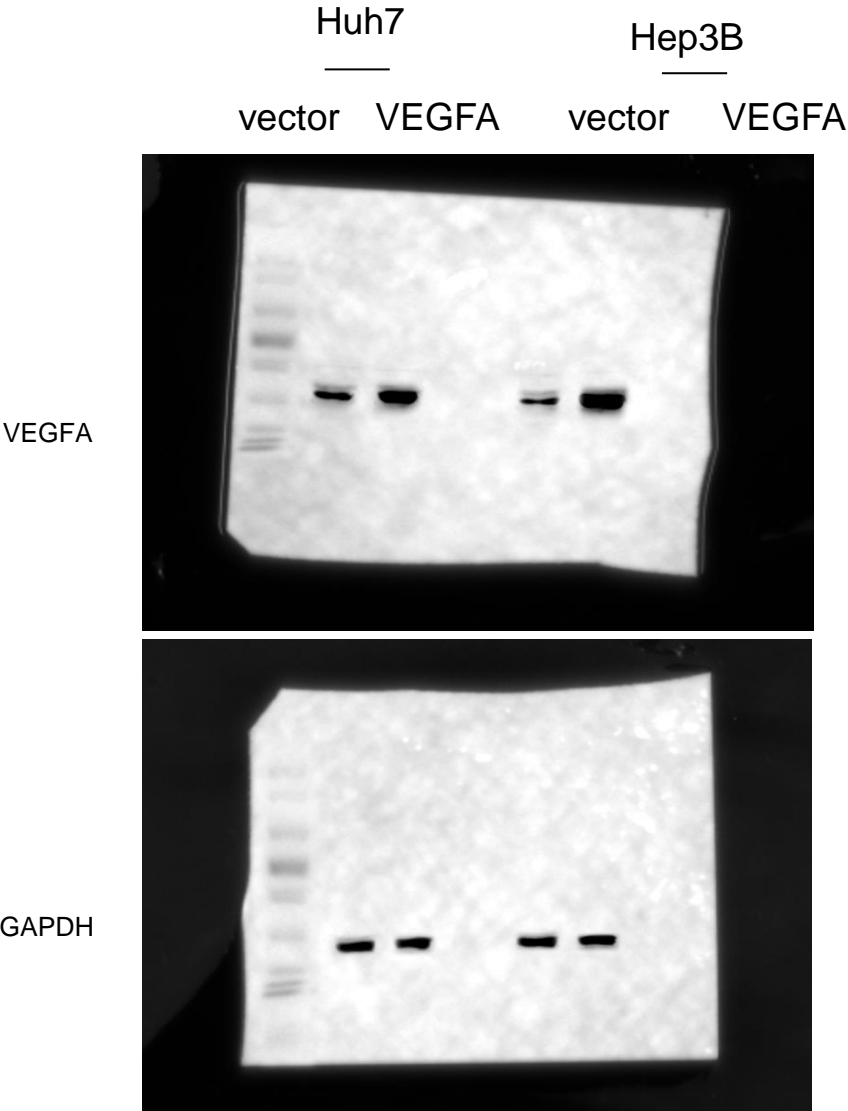

fig7E

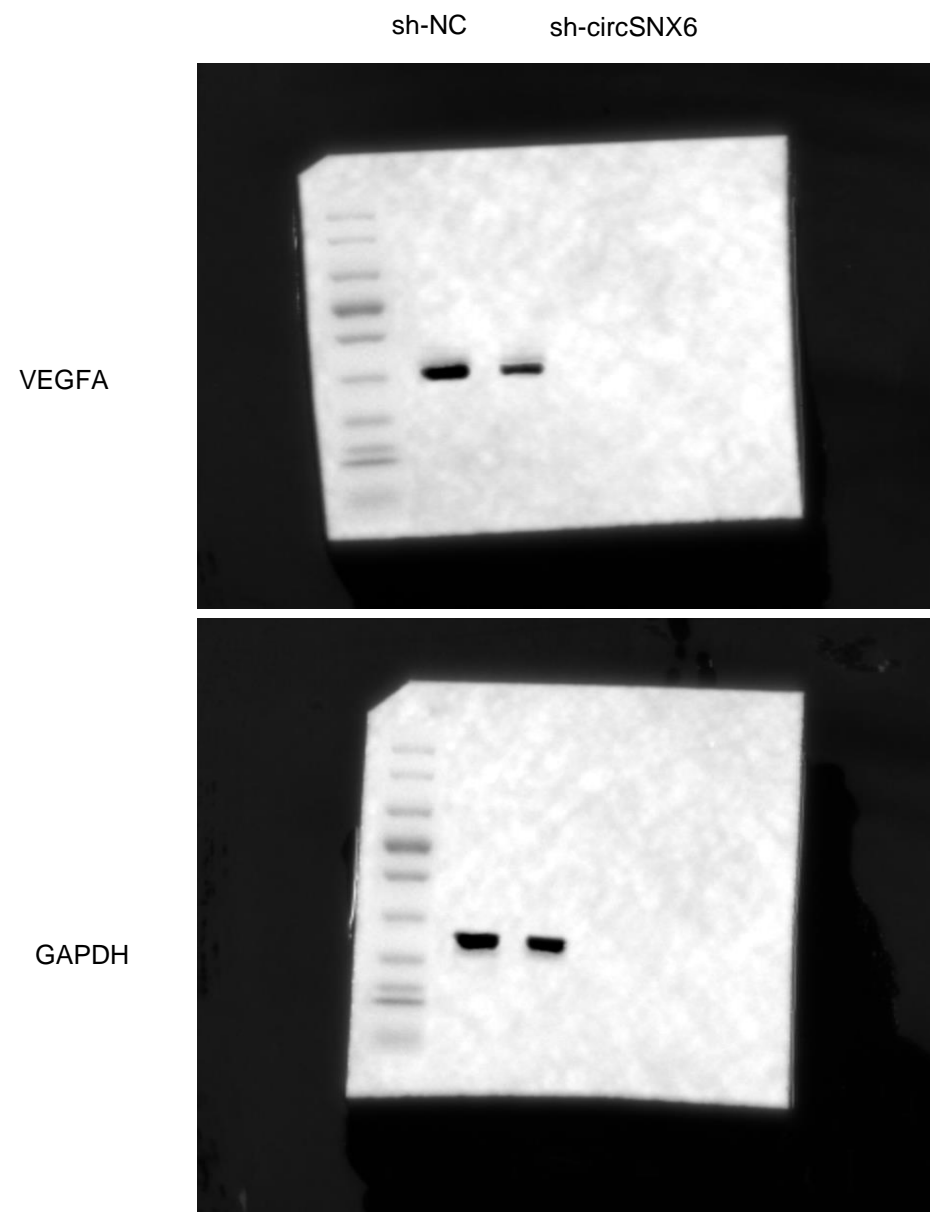

Supplement: Supplementary file 1 — Supplementary Figures. [file 41598_2024_58708_MOESM1_ESM.pdf]
